# Integrated metabolomics and metagenomics reveal plant-microbe interactions driving aroma differentiation in flue-cured tobacco leaves

**DOI:** 10.3389/fpls.2025.1588888

**Published:** 2025-06-03

**Authors:** Yifan Jia, Jianwei Wang, Xiaojie Lin, Taibo Liang, Huaxin Dai, Baojian Wu, Mengmeng Yang, Yanling Zhang, Ruifang Li

**Affiliations:** ^1^ Zhengzhou Key Laboratory of Functional Molecules for Biomedical Research, Henan University of Technology, Zhengzhou, Henan, China; ^2^ College of Biological Engineering, Henan University of Technology, Zhengzhou Henan, China; ^3^ Key Laboratory of Eco-environment and Tobacco Leaf Quality, Zhengzhou Tobacco Research Institute of China National Tobacco Corporation (CNTC), Zhengzhou, Henan, China

**Keywords:** flue-cured tobacco, aroma, untargeted metabolomics, metagenomics, plant-microbe interaction

## Abstract

Current research on tobacco aroma predominantly focuses on single-omics approaches. In this study, we conducted a comprehensive investigation of the relationships between tobacco metabolite profiles, microbial communities, and aroma characteristics. Untargeted metabolomics and metagenomic analyses were performed on flue-cured upper tobacco leaves to compare light aromatic tobacco (LAT) and strong aromatic tobacco (SAT). The results showed that sugar metabolite levels in LAT were significantly higher than those in SAT, whereas levels of specific acids and amino acid metabolites in SAT exceeded those in LAT. Redundancy analysis (RDA) and metabolomic correlation analyses indicated that the genera *Methylorubrum* and *Pseudomonas* may promote sugar metabolite accumulation, while *Pseudokineococcus* potentially regulates both sugar and acid metabolites. In contrast, *Methylobacterium* and *Sphingomonas* were associated with acid and amino acid metabolism, with *Methylobacterium* additionally exhibiting inhibitory effects on sugar metabolism. Metagenomic analysis revealed that *Methylorubrum*, *Pseudomonas*, and *Pseudokineococcus* were abundant in LAT, whereas *Methylobacterium* and *Sphingomonas* dominated in SAT. Notably, the bidirectional regulation of aromatic metabolites by microbial genera such as *Pseudokineococcus* highlights the universality of plant-microbe interactions in shaping metabolic networks—a mechanism potentially applicable to other crop systems. These findings reveal conserved microbial functional traits (e.g., metabolic pathway modulation) that may drive plant phenotypic differentiation beyond tobacco, offering insights into microbiome-mediated crop quality improvement. The results provide theoretical guidance for tobacco aging and aroma regulation and underscore the broader significance of microbial community engineering in agriculture for manipulating plant metabolic outputs.

## Introduction

Plant-microbe interactions play a fundamental role in shaping plant metabolism, secondary metabolite biosynthesis, and overall phenotypic quality across diverse species (Wang et al., 2022). These interactions are driven by complex microbial communities, which participate in biochemical transformations of plant-derived substrates, modulate host metabolic pathways, and influence organoleptic properties (Wang et al., 2022; Shi et al., 2024; Vorholt, 2012). Such mechanisms are pivotal for ecological adaptation and critical for determining the crops’ economic value, as exemplified by tobacco (*Nicotiana tabacum* L.), one of the most studied systems in this context.

Globally, the microbiome contributes to plant fitness through nutrient metabolism (Vorholt, 2012), degradation of complex polymers (e.g., cellulose, starch), and synthesis of volatile organic compounds (VOCs) that define aroma profiles (Lindow and Brandl, 2003; Gong and Xin, 2021). For instance, microbial genera such as *Bacillus*, *Pseudomonas*, and *Aspergillus* are ubiquitous across plant species, where they catalyze the conversion of primary metabolites (e.g., carbohydrates, amino acids) into aromatic precursors via enzymatic activity and metabolic cross-talk with host tissues (Durán et al., 2017). This process is central to the formation of secondary metabolites in many crops, including tea (Li et al., 2018), grapes (Bokulich et al., 2014), coffee (Zhang et al., 2019), aloe vera (Chandel et al., 2025) and tobacco (Huang et al., 2022), where microbial-driven Maillard reactions, protein degradation, and carbohydrate metabolism directly impact sensory attributes (Berg et al., 2014; Banožić et al., 2020).

In tobacco, the interplay between leaf surface microbiota and host biochemistry is particularly evident (Shi et al., 2024). The aroma characteristics of flue-cured tobacco are classified as light, intermediate, or strong aromatic types shaped by microbial-mediated degradation of macromolecules (e.g., proteins, polysaccharides) and subsequent synthesis of flavor-enhancing compounds such as pyrazines and phenolic derivatives. Carbohydrates, phenols, amino acids, organic acids, alcohols, and alkaloids, which are the precursors of tobacco aroma, are the main metabolic compounds (Liu et al., 2022), and carbohydrates are also one of the most important precursors of the tobacco aroma. Study have shown that sugars act as aroma enhancers in the smoke during combustion, producing acids that neutralize the harsh aromas in the smoke, reduce the astringent taste during inhalation, and enhance the overall aromas (Yin et al., 2019). Similar mechanisms have been observed in other aromatic plant, like coffee (Zhao et al., 2024; Wang et al., 2019), where microbial communities regulate the balance of key metabolites (e.g., sugars, organic acids) (Vandenkoornhuyse et al., 2015), ultimately determining product quality. However, the universal principles governing these plant-microbe interactions remain poorly characterized, particularly regarding how the taxonomic and functional diversity of microbiota coordinates with host metabolic networks to drive species-specific phenotypes.

Advancements in multi-omics technologies, including untargeted metabolomics and metagenomics, now empower systematic dissection of these interactions (Diwan et al., 2022). For example, gas chromatography-mass spectrometry (GC-MS)-based metabolomics can reveal conserved metabolic pathways (e.g., carbohydrate degradation, phenylpropanoid biosynthesis) influenced by microbial activity, while microbiome profiling tools (e.g., MetaPhlAn4) elucidate taxonomic shifts linked to functional outcomes. By integrating these approaches, researchers can identify cross-species microbial markers (e.g., *Bacillus subtilis*) that enhance aromatic compound synthesis or mitigate the accumulation of irritants (e.g., ammonia) through nitrogen metabolism regulation (Huang et al., 2022). Such insights transcend individual crops and offer a framework for optimizing microbial consortia in agriculture, fermentation, and post-harvest processing.

This study employs tobacco as a model system to investigate the mechanisms underlying plant-microbe interactions. Using untargeted metabolomics (GC-MS) (Patti et al., 2012), microbiome annotation (MetaPhlAn4) (Segata et al., 2012; Blanco-Míguez et al., 2023), and multivariate analyses, we explore how microbial communities modulate metabolic profiles across different aroma types of flue-cured tobacco, by linking microbial diversity to differential metabolic pathways (Hou et al., 2024).

In this study, we postulate that tobacco aroma is caused by tobacco metabolism and microbial regulation, driving distinct tobacco aroma types. We aim to uncover the conserved principles applicable to broader plant systems, thereby advancing strategies for microbial-driven quality enhancement in crops.

## Materials and methods

### Materials

After collecting from the production area, the fresh upper tobacco leaves were immediately sent to the tobacco curing barn. The flue-cured tobacco leaf samples were collected from the cured tobaccos using aseptic fresh-keeping bags, and sent to laboratory for further research. The four strong aromatic tobacco (SAT) samples were collected from the production areas in Henan province and Hunan province, China. An equal amount of light aromatic tobacco (LAT) samples was collected from the production areas in Sichuan province and Yunnan province, China. Two production areas in each province. One sample from one production area. The detailed information of the samples is described in **Table 1**. The samples were stored at -20°C and returned to room temperature 24 hours before metabolomics and metagenomics experiments. For metabolomics analysis, three replicates for each sample. For metagenomics analysis, one library was performed without replicates.

**Table 1:** Samples’ given names and their detailed information.

Sample names	Cultivar	Aromatic type	Tobacco production area	Province
YNCY	Yun87	light aroma	Yao’an County, Chuxiong City	Yunnan
YNCZ	Yun87	light aroma	Ziwu Town, Yao’an County, Chuxiong City	Yunnan
SCLX	Yun87	light aroma	Xinyun Town, Liangshan Yi Autonomous Prefecture	Sichuan
SCLH	Yun87	light aroma	Huidong County Liangshan Yi Autonomous Prefecture	Sichuan
HeNLL	Zhongyan100	strong aroma	Lingying County, Luohe City	Henan
HeNXY	Zhongyan100	strong aroma	Yulin Town, Xuchang City	Henan
HuNYN	Yun87	strong aroma	Ningyuan County, Yongzhou City,	Hunan
HuNYD	Yun87	strong aroma	Daoxian County, Yongzhou City	Hunan

### Metabolite extraction and derivatization

Referring to the reported method (Liu et al., 2020), the tobacco leaf samples were ground into powder after removing the stems. 20 mg of the tobacco leaf powder was added into 1.5 mL of isopropanol-acetonitrile-water (3:3:2, v/v/v), and sonicated in an ice bath for one hour. After centrifugation at 14,000 rpm for 10 min, 500 μL of the supernatant was transferred into a 1.5 mL injection bottle for vacuum drying. Then 100 μL of 20 mg/mL methoxamine pyridine solution was added and incubated at 37°C, 200 rpm for 90 min. then, 100 μL of N,O-Bis(trimethylsilyl)trifluoroacetamide (BSTFA) was added and incubated at 60°C, 200 rpm for 60 min. The metabolite analysis was performed after the samples were cooled to room temperature.

### GC-MS untargeted metabolomics and metabolic data preprocessing

Metabolomic analysis was performed on an Agilent 5975C instrument (Agilent, USA). Metabolite was separated on a DB-5 MS capillary column (0.25 μm, 0.25 mm × 30 m). The temperature of the injection port was maintained at 300°C. The helium carrier gas flow rate was kept constant at 1.2 mL/min, the injection volume was 1 μL, and the split ratio was 30:1. The mass spectrometer operated in electron impact (EI) mode, and the energy was 70 eV. The detector voltage was kept at 1.2 kV.

The MATLAB high-resolution mass spectrometry data analysis toolkit was used to perform baseline correction, peak extraction, annotation, and alignment of the collected metabolic data. Relative quantification of metabolites was performed using area normalization.

### Statistical analysis of metabolomics data

Metabolites were characterized and identified referring to the MS spectral database library (NIST v2.3, https://chemdata.nist.gov/dokuwiki/doku.php?id=chemdata:nist17). Data processing and graphing were performed using Prism. Partial least squares discriminant analysis (PLS-DA) was performed on the data using R language (v4.4, https://cloud.r-project.org/) (Xia and Sun, 2022). PLS-DA reduces the dimensionality of the data and performs discriminant analysis on regression results with specific discriminative thresholds by combining a regression model.

The R^2^ coefficient quantifies the proportion of variance in the data that the model explains. A higher R^2^ value indicates a better fit between the model and the data. Q^2^ is an indicator of the predictive ability of a model. A higher Q^2^ value indicates better predictive performance, and R^2^ should be greater than Q^2^ (Blaise et al., 2021). Metabolites were ranked according to the contribution of each component (Variable Importance in Projection, VIP) to the PLS-DA model. VIP>1 was used as the threshold. Metabolites that reached the threshold were considered differential metabolites. MetaboAnalyst 5.0 (https://www.metaboanalyst.ca/) analyzed the kyoto encyclopedia of genes and genomes (KEGG) pathway enrichment of differentially expressed metabolites. A significance threshold of P ≤ 0.05 was used to obtain the results of significantly enriched metabolic pathways, and final illustrations were refined using Adobe Illustrator (Pang et al., 2024).

### Genomic library construction, sequence and data preprocessing

Shotgun metagenomics sequencing method was performed by Novogene Co. Ltd (Beijing, China). The total genomic DNA from tobacco leaf samples was extracted using the Magnetic Plant Genomic DNA Kit (Tiangen, China) following the manufacturer’s instructions. All operations of DNA extraction were carried out in a sterile environment. The genomic DNA purity and integrity were checked by 1% agarose gel electrophoresis. The genomic DNA was randomly sheared into short fragments of approximately 350 bp. The DNA fragments were subjected to end-repaired, A-tail and further ligated with Illumina adapters. The quantitative real-time polymerase chain reaction method was then used to quantify the effective concentration of the library (>3 nM) to ensure its quality. The quantified library was pooled and sequenced on Illumina Novaseq6000 (Illumina, USA), producing 2 × 150 bp paired-end reads. Metagenomic data were quality-controlled and trimmed for adaptors using fastp (https://github.com/OpenGene/fastp). Considering the possibility of host contamination in samples, Bowtie2 software (http://bowtie-bio.sourceforge.net/bowtie2/index.shtml) filter out reads that may come from host origin. Taxonomic profiling of the filtered sequence data was performed using MetaPhlAn4 (Blanco-Míguez et al., 2023).

### Statistical analysis of metagenomic

The alpha diversity indices, including ACE, Chao1, Shannon, and Simpson, were calculated by Mothur (https://mothur.org/wiki/calculators/). Alpha diversity is mainly used to study the diversity of microbial communities in a sample and is evaluated using a series of alpha diversity indices to obtain microbial information such as microbial species richness and diversity. R was used for ANOSIM (Analysis of similarities), beta diversity, and RDA analysis. ANOSIM is a non-parametric test method based on permutation and rank sum tests. The obtained R-value represents the relationship of the intergroup and intragroup differences. In this study, the ANOSIM on the genus level was performed. Beta diversity is used to describe the inter habitats variation in biological communities. RDA analysis (Redundancy analysis) is an environmental factor-constrained PCA analysis mainly used to explore the relationship between community species composition and environmental variables. This study used genus-level microorganisms as environmental factors to explore the relationship between metabolites and microbial communities. Galaxy 2.0 (http://galaxy.biobakery.org/) was used for LEfSe analysis to screen biomarkers with significant differences between groups, detecting the different species of the subgroups using the rank sum test and downscaling and evaluating the effect size of the different species or functions using LDA (Linear Discriminant Analysis). The SPSS (v29.0, https://www.ibm.com/spss) software for correlation analysis was used to calculate coefficients and significance, with the R for visualization. Correlation analysis is the process of analyzing two or more correlated variables to measure the closeness of the correlation between two variables.

## Results

### Metabolite composition in flue-cured tobacco leaves

**Figure 1:**
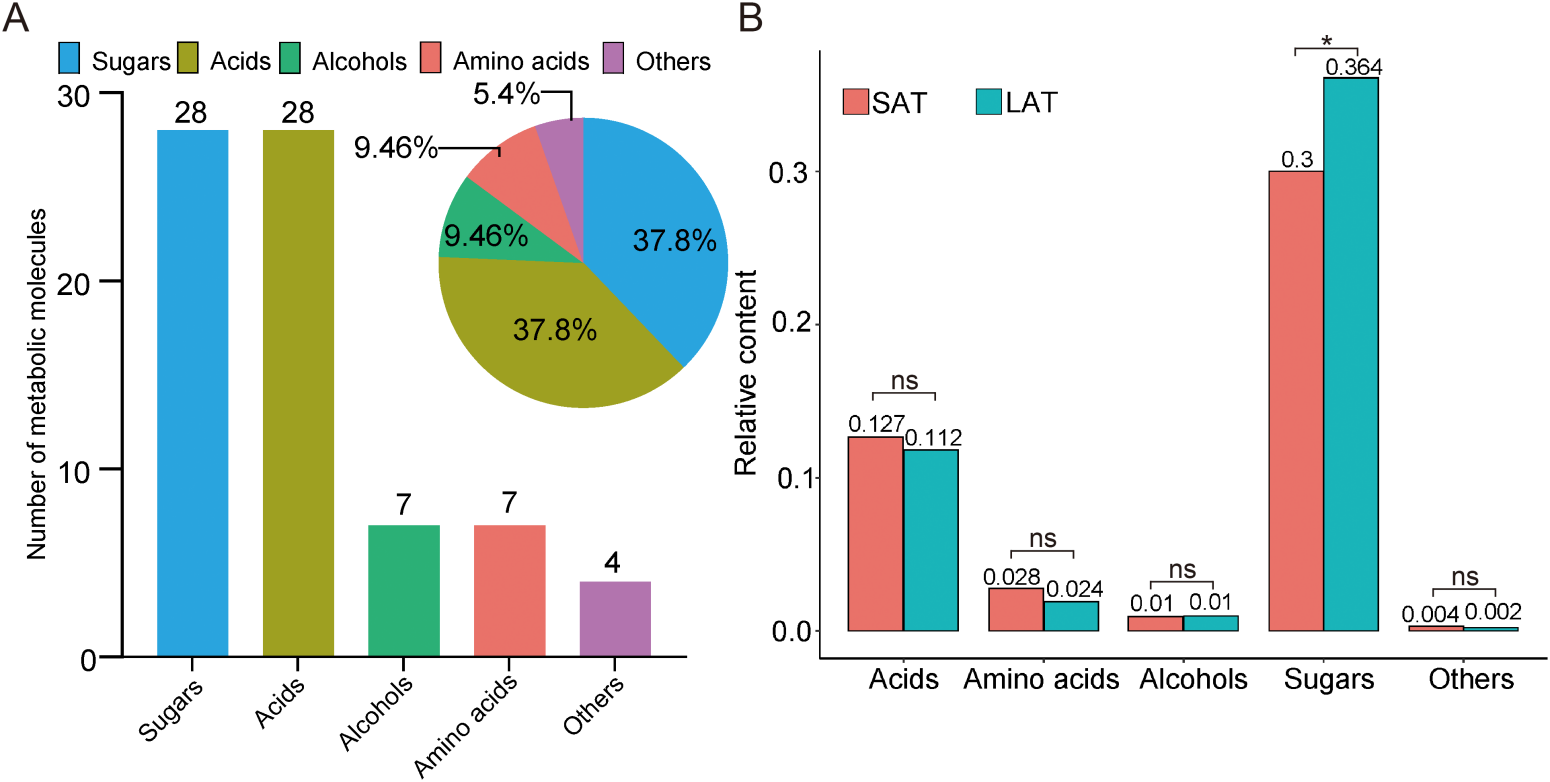
Untargeted metabolomics data of tobacco from GC-MS. **(A)** Metabolic molecule (Bar plot) and their proportion (Pie plot). **(B)** The relative contents of metabolites in different aromatic flue-cured tobacco. *P<0.05, ns, no significance. SAT, strong aromatic tobacco; LAT, light aromatic tobacco.

A total of 74 small metabolites were identified (with a matching score >700 in the NIST database). Based on their structural and chemical characteristics, these metabolites were classified into four categories: 28 sugars, 7 alcohols, 7 amino acids, and 28 acids (**Figure 1A**). The relative contents of tobacco metabolites in the flue-cured upper tobacco leaves are shown in **Table 2**. A bar chart of the relative contents of the different aromatic types is shown in **Figure 1B**. The relative contents of sugars in all samples were the highest, while the relative contents of acids were much lower than those of sugars. The relative contents of sugar compounds in SAT samples were significantly lower than those in LAT samples. The relative contents of amino acids and acids in SAT samples were higher than those in LAT but not significant. The relative contents of alcohols showed no significant differences between SAT and LAT samples. The results demonstrated that sugars, acids, and amino acids may be the main effectors on the aromas of the flue-cured tobacco. Sugars are positively correlated with light aromas of tobacco. They are negatively correlated with strong tobacco aromas, while the effects of acids and amino acids on tobacco aromas were inversely related to sugars.

**Table 2:** Relative content of metabolites in different tobacco samples.

Metabolite type	HeNLL	HeNXY	HuNYD	HuNYN	SCLX	SCLH	YNCZ	YNCY
Amino acids	0.035	0.038	0.017	0.02	0.02	0.02	0.028	0.022
Acids	0.099	0.101	0.132	0.174	0.140	0.123	0.127	0.059
Alcohols	0.008	0.01	0.01	0.01	0.009	0.011	0.008	0.009
Sugars	0.328	0.34	0.261	0.27	0.349	0.354	0.356	0.4
Others	0.004	0.003	0.004	0.004	0.004	0.002	0.002	0.002

### Bioinformatics characteristics of metabolites in different aroma types of flue-cured tobacco leaves

**Figure 2:**
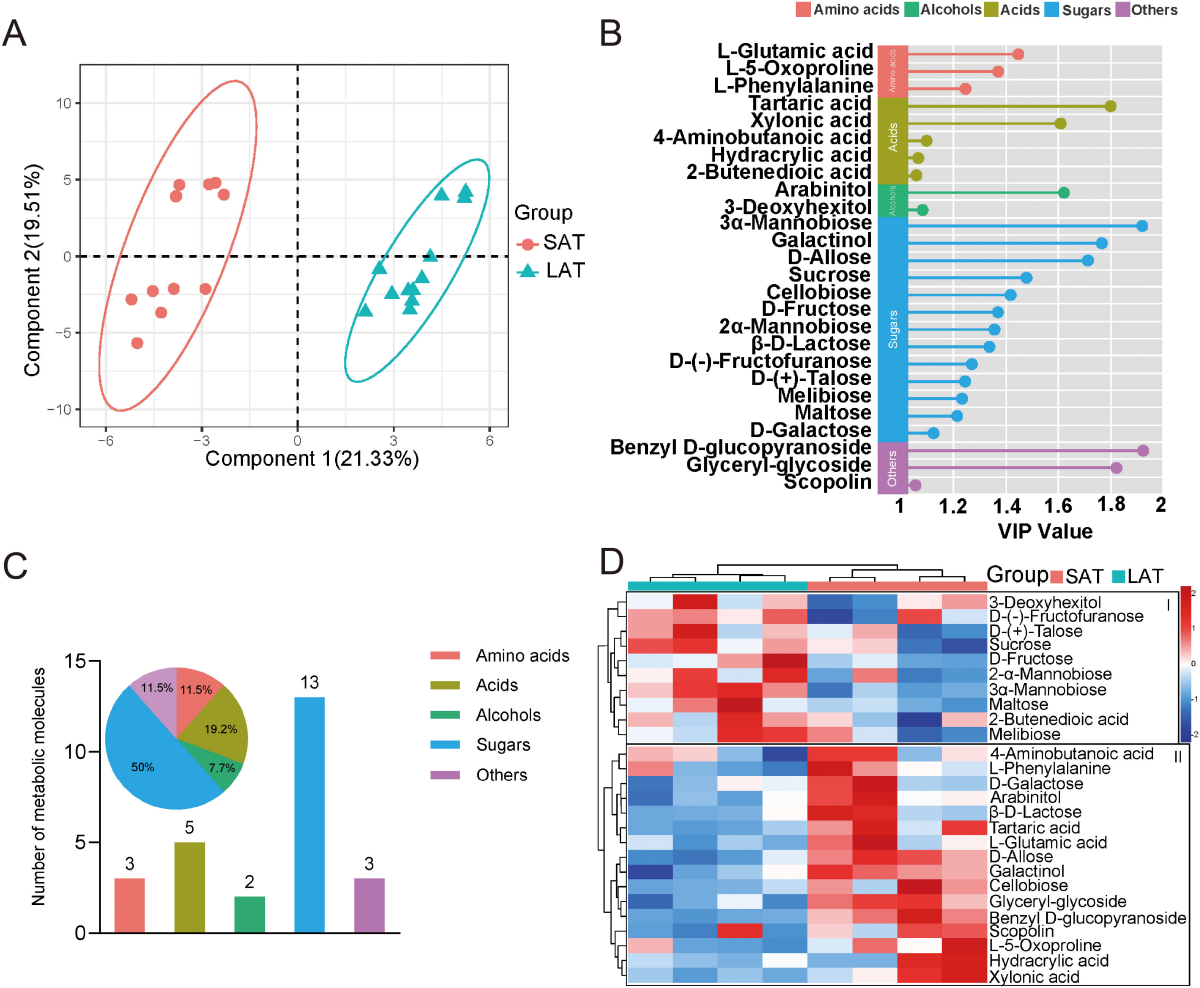
Multivariate analysis of the aromatic flue-cured tobacco leaf metabolites. **(A)** PLS-DA score plot, ellipses represent 95% confidence intervals for each group. **(B)** Dot plot of metabolites with variable importance in projection (VIP) scores >1.0 in the PLS-DA model. **(C)** Metabolite composition, bar plot showing the number of the metabolite molecules, pie plot displaying the proportion of metabolites. **(D)** Heat map of differential metabolites relative content in tobacco leaves with different flavors (Euclidean distance). SAT, strong aromatic tobacco; LAT, light aromatic tobacco.

Multivariate analysis of metabolic profiles was conducted through PLS-DA to characterize aromatic differentiation in flue-cured tobacco leaves (**Figure 2A**). In the PLS-DA analysis, the first two principal components (PCs) explained 40.8% of the total variance, with PC1 and PC2 explaining 21.3% and 19.5%, respectively. The samples were clearly separated in the score plot. The R^2^ and Q^2^ values in PLS-DA were 0.99 and 0.96, respectively, indicating the model has a high stability and predictive ability. The results of the score plot exhibited a good separation between LAT and SAT.

**Figure 3:**
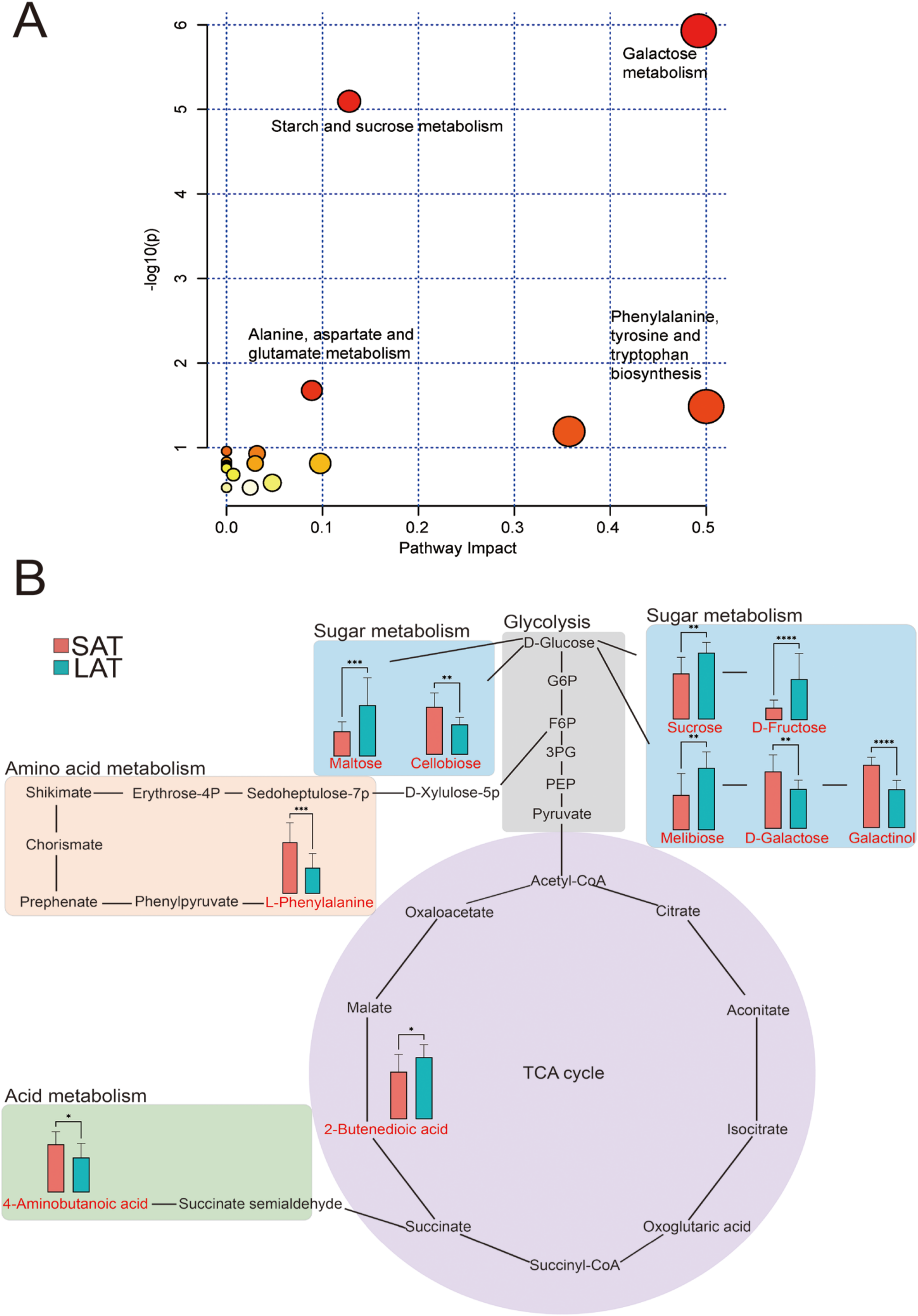
Metabolome analysis of differential metabolites in light and strong aromatic tobacco leaves. **(A)** Overview of metabolic pathways. **(B)** Metabolic pathway map of the differential metabolites. *P<0.05, **P<0.01, ***P<0.001, ****P<0.0001. In **(A)**, the bubbles represent the metabolic pathways, and the color of the bubbles (from yellow to red) indicates the significant level of the metabolites in the data.

The metabolites’ relative importance value (VIP) with the projection variable greater than 1 as the threshold for differential metabolites, a total of 26 characteristic biomarkers were found (**Figure 2B**). After classifying, sugar metabolites were still the most numerous, indicating that sugar, as the main differential metabolites, play an important role in the metabolic regulation of the tobacco aromatic type (**Figure 2C**). Subsequently, the heat map of the characteristic metabolites is shown in **Figure 3D**. All the differential metabolites abundance can be divided into two categories (**Figure 2D**), group I and group II, according to their different types. In group I, the relative contents of most sugar metabolites, such as Sucrose, Fructose, Maltose, were higher in the LAT samples. In comparison, the relative contents of most acids and amino acids metabolites, such as L-Phenylalanine, L-5-Oxoproline, Hydracrylic acid, and Xylonic acid, in group II were higher in the SAT samples. Among them, the contents of sugar metabolites, such as 3α-Mannobiose and Maltose, in the LAT subgroup were higher than those in the SAT subgroup, indicating that sugar metabolites are important LAT biomarkers. In contrast, amino acids, such as L-5-Oxoproline and L-Phenylalanine, as well as acids metabolites, such as Hydracrylic acid and Xylonic acid, have high contents in the SAT subgroup, indicating that amino acids and acids metabolites may be SAT biomarkers. Notably, the total amino acid content in SAT samples from HuNYD and HuNYN was notably lower than that from HeNLL and HeNXY. Despite this regional variation, L-phenylalanine, a key aromatic precursor, showed elevated levels in three out of four SAT samples compared to their LAT counterparts. The exception was HuNYD, where L-phenylalanine content was marginally lower in SAT, possibly due to site-specific environmental stressors. These findings suggest that L-phenylalanine accumulation in SAT is not universally consistent across regions but may still contribute to aroma profiles when upregulated.

### Metabolic pathway of flue-cured tobacco leaves

In order to gain an in-depth understanding of the differences in the metabolic networks between the SAT and LAT samples, the metabolic pathways of all differential metabolites were analyzed using MetaboAnalyst 5.0, and 12 differential metabolites were involved in 17 metabolic pathways (**Table 3**). The metabolic pathways with a p-value less than 0.05 were selected as differential ones; 10 differential metabolites were enriched in 4 differential metabolic pathways. The metabolic pathways in **Figure 3** showed the overall situation of the differential metabolites in the tobacco leaf samples. Four metabolic pathways (glycolysis, sugar metabolism, acid metabolism, amino acid metabolism, and tricarboxylic acid cycle (TCA) were extracted and connected according to the KEGG pathway database. Most of the differential metabolites were concentrated in the pathways related to sugar metabolism. In the SAT samples, the relative contents of differential metabolite related to amino acid metabolism were significantly higher than those in the LAT. In comparison, the relative contents of most sugar metabolites, such as sucrose, fructose, and maltose in the LAT samples were significantly higher than those in the SAT.

**Table 3:** Metabolic pathways of differential metabolites in tobacco leaves.

Pathway Name	Total	Expected	Hits	Raw p	Impact	Hit Metabolites
Galactose metabolism	27	0.22286	5	1.18E-06	0.49222	Sucrose
						Melibiose
						D-Galactose
						D-Fructose
						Galactinol
Starch and sucrose metabolism	18	0.14857	4	8.03E-06	0.12786	Sucrose
						Maltose
						Cellobiose
						D-Fructose
Alanine, aspartate and glutamate metabolism	28	0.23111	2	0.021069	0.08894	4-Aminobutanoic acid
						2-Butenedioic acid
Phenylalanine, tyrosine and tryptophan biosynthesis	4	0.033016	1	0.03264	0.5	L-Phenylalanine
Phenylalanine metabolism	8	0.066032	1	0.064294	0.35714	L-Phenylalanine
Arginine biosynthesis	14	0.11556	1	0.10999	0	2-Butenedioic acid
Butanoate metabolism	15	0.12381	1	0.1174	0.03175	4-Aminobutanoic acid
Pentose and glucuronate interconversions	19	0.15683	1	0.14648	0	Arabinitol
Tricarboxylic acid cycle (TCA)	20	0.16508	1	0.15361	0.02981	2-Butenedioic acid
Fructose and mannose metabolism	20	0.16508	1	0.15361	0.09765	D-Fructose
beta-Alanine metabolism	21	0.17333	1	0.16069	0	Hydracrylic acid
Propanoate metabolism	22	0.18159	1	0.16771	0	Hydracrylic acid
Pyruvate metabolism	23	0.18984	1	0.17468	0	Hydracrylic acid
Glutathione metabolism	28	0.23111	1	0.20871	0.00709	Hydracrylic acid
Arginine and proline metabolism	36	0.29714	1	0.26048	0.04767	Hydracrylic acid
Amino sugar and nucleotide sugar metabolism	42	0.34667	1	0.29724	0	Hydracrylic acid
Tyrosine metabolism	42	0.34667	1	0.29724	0.02463	Hydracrylic acid

### Microbial community alpha diversity of flue-cured tobacco leaves

After cleaning the reads of the tobacco leaf sample, species annotation was performed using MetaPhlAn; 68 microbial species were detected in the LAT and SAT samples. The ACE, Chao1, Shannon, and Simpson indices reflect the number of communities, species abundance, species diversity, and species evenness in the sample (Zhang et al., 2022). The α diversity index is shown in **Supplementary Table S1**. The ACE index, Chao1, Shannon, and Simpson indices of SAT were higher than those of LAT, indicating that the SAT leaves contain more microbial species than those of LAT, and the microbial composition was even.

### Microbial community beta diversity of flue-cured tobacco leaves

Beta diversity is used as a measure of the variation in species diversity. The principal coordinates analysis (PCoA) of the Bray-Curtis distance on the species level is shown in **Supplementary Figure S1A**. The SAT and LAT samples were significantly separated, and the two principal coordinate axes, PCo1 and PCo2, explain 63.08% of the total variance. The differences in microbial communities between SAT and LAT samples were apparent. Hierarchical clustering divided the flue-cured tobacco leaves from different production areas into two branches (**Supplementary Figure S1B**). One branch contained all tobacco leaf samples from Henan and Hunan provinces, which are SAT leaf samples. The other branch contained tobacco leaf samples from Sichuan and Yunnan which are LAT leaf samples. It is worth noting that only one species, *Aspergillus_flavus*, was found in the species annotation of the SCLH, an LAT leaf sample in MetaPhlAn, indicating that *Aspergillus_flavus* may be the abundant species among the microorganisms on the surface of the tobacco leaves from Liangshan Yi Autonomous Prefecture in Huidong County, Sichuan Province.

### Characteristics of microbial communities of flue-cured tobacco leaves

The Anosim result at the genus level is shown in **Supplementary Figure S2A**, where R>0 and p<0.05, demonstrating significantly greater inter-group variation compared to intra-group differences. Significant differences existed in the aromatic types and relative abundances of microbial genera between SAT and LAT.

The distribution of the microbial composition at the phylum level of SAT and LAT samples was shown in **Supplementary Figure S2B**. Five bacterial phyla and one fungal phylum were detected at the phylum level. Five bacterial phyla were *Proteobacteria*, *Actinobacteria*, *Bacteroidetes*, *Firmicutes*, and *Deinococcus_thermus*; one fungal phylum was *Ascomycota*. Among them, *Proteobacteria*, *Actinobacteria*, and *Bacteroidetes* were the main abundant taxa in the SAT samples (relative abundance > 1%), while in the LAT samples, *Proteobacteria* was the abundant bacterial phylum and *Ascomycota* was the abundant fungal phylum. *Proteobacteria* was the abundant t bacterial phylum in both types of aromatic tobacco leaves, with a relative abundance of 95% and 72% of the total microbial community in the SAT and LAT samples, respectively. In contrast, the relative abundance of the fungal phylum *Ascomycota* in the LAT leaves (27.37%) was higher than that in the SAT samples (0.29%). The relative abundance of the bacterial phylum *Actinobacteria* in the SAT samples (2.7%) was higher than that in the LAT samples (0.005%).

At the genus level (**Supplementary Figure S2C**), ten bacterial genera and one fungal genus (relative abundance >1%) were identified. Among them, *Pseudomonas*, *Sphingomonas*, *Methylobacterium*, *Methylorubrum*, *Aureimonas*, *Enterobacter*, *Pseudokineococcus*, *Spirosoma*, *Pantoea*, and *Afipia* were the main abundant bacterial genera. *Aspergillus* was the main abundant fungal genus. The composition and relative abundance of the main bacterial and fungal genera differed. The relative abundance of *Sphingomonas* (57.5%) in the SAT group was significantly higher than that in the LAT group (23%), the relative abundance of *Aspergillus* (27.4%), *Pseudomonas* (36.1%), and *Methylorubrum* (9.7%) in the LAT group was higher than that in the SAT group (0.3%, 9.2%, and 2.5%). *Methylobacterium* (23.5%), *Aureimonas* (1.2%), *Spirosoma* (1.2%), and *Pseudokineococcus* (1.9%) were only found in the SAT group. In comparison, the genus *Pantoea* (1.1%) was only found in the LAT group.

**Supplementary Figure S2D** shows the LEfSE analysis of the LAT samples and SAT samples at the genus level. Three bacterial genera were identified as differential abundant species between the two tobacco groups based on the threshold of greater than 4 in LDA score and less than 0.05 in P value.

Overall, the two types of aromatic samples showed species differences and microbial communities’ abundance at the phylum and genus levels. Combined with the results of the LEfSe analysis, the differences in microbial communities between the two types of aromatic samples were mainly reflected in the differences in the abundance of *Methylobacterium*, *Pseudokineococcus*, and *Quadrisphaera*, which may potentially influence tobacco aroma development.

### Association of abundant microorganisms and metabolites of flue-cured tobacco leaves

**Figure 4:**
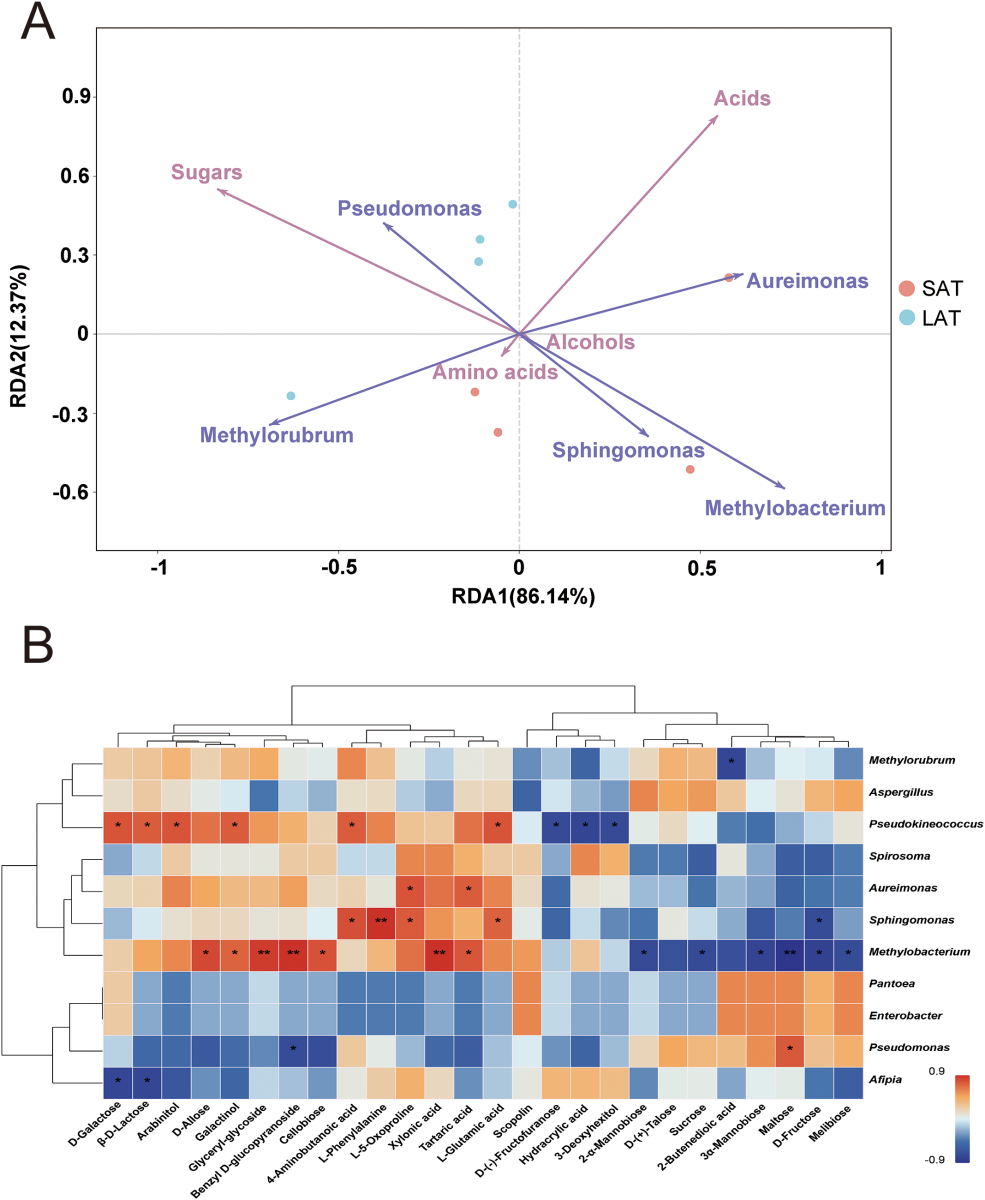
Metabolites and microorganisms of the flue-cured tobacco leaves. **(A)** RDA analysis, the angle between two variables less than 90° indicates a positive correlation, otherwise a negative correlation. **(B)** Correlation analysis between microorganisms at the genus level and metabolites. *P<0.05, **P<0.001.

Redundancy analysis (RDA) of the influence of the abundant microbial genera on metabolites showed that the first two axes RDA1 and RDA2 collectively explained 98.51% of the variation in the relationship between metabolites and microorganisms of the samples, with RDA1 contributing 86.14% and RDA2 12.37% (**Figure 4A**), indicating that RDA1 is the main dimension that distinguishes the relationship between metabolites and microbial communities in LAT and SAT samples. In the statistical validation identified, the effects of the microbial genera *Methylobacterium* and *Methylorubrum* on metabolites were significant, with p-values of 0.003 and 0.035, respectively, indicating that they are key genera in the microbial community that regulate the metabolites distribution and cause differences in the aromas of tobacco. Sugar metabolites associated with light aromas showed positive correlations with Methylorubrum (RDA1=-0.691) and Pseudomonas (RDA1=-0.375) but negative correlations with Methylobacterium (RDA1 = 0.733). Conversely, acid metabolites associated with strong aromas, were positively correlated with the genera *Methylobacterium* and *Aureimonas* (RDA1 = 0.733 and 0.619) and negatively correlated with the genera *Methylorubrum* and *Pseudomonas* (RDA1 = -0.691 and -0.375). In addition, amino acids, which are also important for tobacco to form strong aromas, were positively correlated with the genus *Sphingomonas* (RDA2 = -0.736) and negatively correlated with the genera *Aureimonas* (RDA2 = 0.228).

The distribution of the metabolites and the superior microbial genus (relative abundance >1%) showed that the genus *Methylobacterium* was closely related to strong aromas, indicating that acids and amino acids dominate its metabolic characteristics; the genus *Methylorubrum* and *Pseudomonas* were closely related to light aromas, indicating that sugars dominated its metabolic characteristics. This result suggested that the selective regulation of metabolites by different abundant genera in the microbial community may influence the aromatic types.

From the analysis results, the genus *Methylobacterium* and *Methylorubrum* are the core genus in regulating the LAT and SAT metabolites, and their contribution to the metabolism of acids, amino acids, and sugars are particularly significant. The association analysis between abundant microorganisms and metabolites is shown in **Figure 4B**. Combined with the RDA analysis results, the microbial genus *Methylobacterium* was significantly positively correlated with the acid metabolites Xylonic acid and Tartaric acid and also significantly negatively correlated with most of the sugar metabolites, such as Sucrose, Maltose, and D-Fructose. The results indicated that *Methylobacterium* may play an important role in the overall metabolic regulation of the sample by promoting acid metabolism and inhibiting the accumulation of sugar metabolites, resulting in a strong aroma of tobacco.

The genus *Sphingomonas* is significantly positively correlated with the acid metabolite 4-Aminobutanoic acid, as well as the amino acid metabolites L-Phenylalanine, L-Glutamic acid, and L-5-Oxoproline, negatively correlated with the sugar metabolite D-Fructose. The results indicated that the genus *Sphingomonas* plays a facilitating role in amino acid and acid metabolism while inhibiting some sugar metabolism and resulting in a strong tobacco aroma.

The genus *Pseudokineococcus* was significantly positively correlated with the sugar and acid metabolites D-Galactose, β-D-Lactose and 4-Aminobutanoic acid, also significantly negatively correlated with D-(-)-Fructofuranose and Hydracrylic acid, indicating that the genus *Pseudokineococcus* plays an important role in the formation of the metabolites related to tobacco aroma. From the results of species abundance at the genus level (**Supplementary Figure S2C**), *Pseudokineococcus* existed more in LAT than in SAT, illustrating that it may play an important role in sugar metabolism and results in a light aroma of tobacco.

In summary, the complex regulation between abundant microbial genera and metabolites was clarified through redundancy analysis (RDA) and correlation analysis. The genus *Methylobacterium* played a key role in promoting acid metabolism and inhibiting sugar metabolism, highlighting its central position in distinguishing the metabolic characteristics of tobacco. The genera *Methylorubrum* and *Pseudomonas* were mainly involved in the sugar metabolic pathway, promoting the accumulation of sugar metabolites in the LAT sample, while the main function of the genus *Sphingomonas* was promoting the amino acid and some acid metabolic pathways, which benefit strong aromas formation. In addition, the bidirectional regulation of sugars and acid metabolites by the genus *Pseudokineococcus* further reflects its important role in the metabolic network. These results revealed that different abundant microbial genera jointly drive the metabolic differences between the two types of aromatic tobacco samples by selectively regulating the distribution of metabolites.

## Discussion

This study revealed significant differences and the association of the metabolite composition and microbial communities of the flue-cured tobacco leaves of the light aroma and strong aroma by combining analysis of untargeted metabolomics and metagenomics results. The results showed that tobacco leaves with different aromas exhibited significant differences in metabolites distribution and were regulated by specific abundant microbial genera. Importantly, the findings align with broader principles of plant-microbe interactions observed across plant species, offering insights into evolutionary conserved mechanisms and functional modularity in microbial communities (de Vries et al., 2020; Frantzeskakis et al., 2020; Mesny et al., 2023).

The composition and content of the metabolites in tobacco are highly consistent with the sensory characteristics of tobacco aroma (Zhang et al., 2024). The high content of sugar metabolites gives the tobacco a soft aroma by flavoring and reducing the pungent taste. In contrast, the rich acids and amino acids enhance the depth and complexity of the tobacco aromas by participating in the Maillard reaction and other chemical reactions (Geng et al., 2023). In the present study, the metabolite analysis showed that sugar and acid metabolites were the main contributors to tobacco aromas, manifesting the metabolic differences between LAT and SAT samples. The LAT sample significantly contained higher levels of sugar metabolites (e.g., sucrose, maltose) than the SAT sample, consistent with the findings of Jing et al (Jing et al., 2024). Conversely, the SAT samples contained higher acid metabolites (e.g., xylonic acid, tartaric acid) and amino acid metabolites (e.g., L-5-Oxoproline, L-Glutamic acid). However, in the study by Tie et al (Tie et al., 2024), LAT tobacco leaves had a higher content of amino acids, which contradicts our conclusion. This discrepancy may arise from differences in tobacco varieties or cultivation practices. Notably, such context-dependent metabolic variations are also observed in Arabidopsis-microbiome studies, where host genotype and environmental factors significantly reshaped microbial functional outputs (Beck et al., 2022; Gonçalves et al., 2023). However, unlike the simplified model system of Arabidopsis, tobacco’s complex secondary metabolism and agricultural management introduce additional layers of microbial community regulation.

Microorganisms indirectly affect tobacco leaves’ aroma by modulating metabolites’ types and contents. *Sphingomonas* degrades dimeric lignin compounds into flavor precursors, while *Methylobacterium* utilizes methanol to drive one-carbon metabolism. *Pseudomonas* participates in nicotine degradation, redirecting intermediates into the TCA cycle. These functional roles reflect the “core-accessory” framework of microbial metabolic networks, where core functions (e.g., carbon/nitrogen cycling) are conserved across plant species, while accessory functions (e.g., specialized aroma synthesis) are niche-specific (Mazurie et al., 2010; Roume et al., 2015; Ramon and Stelling, 2023). For instance, *Methylobacterium*’s methanol metabolism is a core trait in both tobacco and Arabidopsis phyllosphere communities, but its contribution to aroma formation is uniquely amplified in tobacco due to host-specific secondary metabolism (Zhang et al., 2020; Zheng et al., 2024). Evolutionarily, such conservation suggests that plant-microbe co-adaptation in agricultural systems builds upon ancient symbiotic mechanisms repurposed for crop-specific traits (de Vries et al., 2020).

The identification of *Quadrisphaera* as a differentially abundant taxon in the LEfSe analysis despite its low relative abundance (0.12%) highlights the interplay between statistical significance and biological relevance in microbiome studies. The possible explanations are listed as below: (1) LEfSe identifies taxa with significant differences in relative abundance between groups by combining non-parametric tests with effect size estimation (LDA score). In this study, *Quadrisphaera* was exclusively detected in SAT group but absent in LAT group, leading to statistically significant differences (P < 0.05) even at low abundance. LEfSe’s non-parametric approach is sensitive to such categorical differences, especially when taxa are uniquely associated with a group. (2) Microbial communities often include rare taxa that contribute disproportionately to functional processes. Although *Quadrisphaera*’s abundance was low, its metabolic activity (e.g., niche-specific enzymatic functions) or interactions with other microbes could amplify its ecological impact. For instance, *Quadrisphaera* might participate in pathways influencing secondary metabolite synthesis or niche competition, indirectly shaping aroma differentiation. Anyway, the biological significance of Quadrisphaera in this context requires further validation (e.g., strain isolation, functional assays). However, its identification aligns with emerging evidence that rare taxa can serve as biomarkers in plant-microbe systems. Future studies with larger sample sizes and metatranscriptomics could clarify its role.

Cross-species comparisons further highlight conserved metabolic pathways shaped by microbial activity. For example, phenylpropanoid biosynthesis-a pathway critical for aroma in tobacco, tea, and grapes—is similarly modulated by microbial hydroxylation and methylation enzymes in diverse plants (Zhao et al., 2020, 2023; Wang et al., 2023). In our study, the enrichment of sugar degradation pathways in LAT tobacco mirrors microbial-driven carbohydrate metabolism in maize rhizosphere communities, underscoring a universal strategy for balancing carbon allocation and secondary metabolite synthesis (Thoenen et al., 2023). These parallels emphasize and also support our hypothesis that microbial functional redundancy and metabolic flexibility are key drivers of plant phenotypic diversity.

Although amplicon sequencing (e.g., 16S/ITS rRNA gene sequencing) is widely recognized as a cost-effective and standardized approach for taxonomic profiling, in this research, shotgun metagenomic sequencing was selected over amplicon sequencing to (i) avoid PCR amplification biases, and (ii) integrate taxonomic data with metabolomic profiles for holistic plant-microbe interaction modeling.

## Conclusion and prospects

On the flue-cured tobacco leaves with a light aromatic smell, the abundant genera *Methylorubrum* and *Pseudomonas* promote sugar metabolism, while the genus *Pseudokineococcus* exhibits a bidirectional regulation of aromatic metabolites. While on the flue-cured tobacco leaves with a strong aromatic smell, the abundant genera *Methylobacterium* and *Sphingomonas* promote the metabolism of acids and amino acids. Meanwhile, the genus *Methylobacterium* inhibits sugar metabolism. This study provides a theoretical basis for improving tobacco leaf quality through metabolomic and metagenomic analysis. However, a limitation of this study is the absence of direct functional annotation of microbial genes (e.g., KEGG pathways). Future work combining metatranscriptomics or MAG-based approaches with controlled experiments (e.g., gnotobiotic systems) will validate the hypothesized roles of taxa like *Methylobacterium* in sugar/acid metabolism. Meanwhile, evolutionary perspectives (e.g., phylogenetic conservation of microbial traits) and multi-omics modeling should also be integrated in the future research to dissect how core-accessory microbial functions coevolve with host metabolic networks. Additionally, exploring the molecular mechanisms of microbial-metabolite interactions in tobacco could inform microbiome engineering strategies applicable to other crops, such as enhancing stress tolerance or flavor profiles through targeted microbial consortia.

## Data Availability

The raw data supporting the conclusions of this article will be made available by the authors, without undue reservation.
