# Supplementary figures and images for: Integrated metabolomics and metagenomics reveal plant-microbe interactions driving aroma differentiation in flue-cured tobacco leaves
Source: Front Plant Sci. 2025 Jun 3;16:1588888. doi: 10.3389/fpls.2025.1588888 (PMC12170567; doi:10.3389/fpls.2025.1588888)

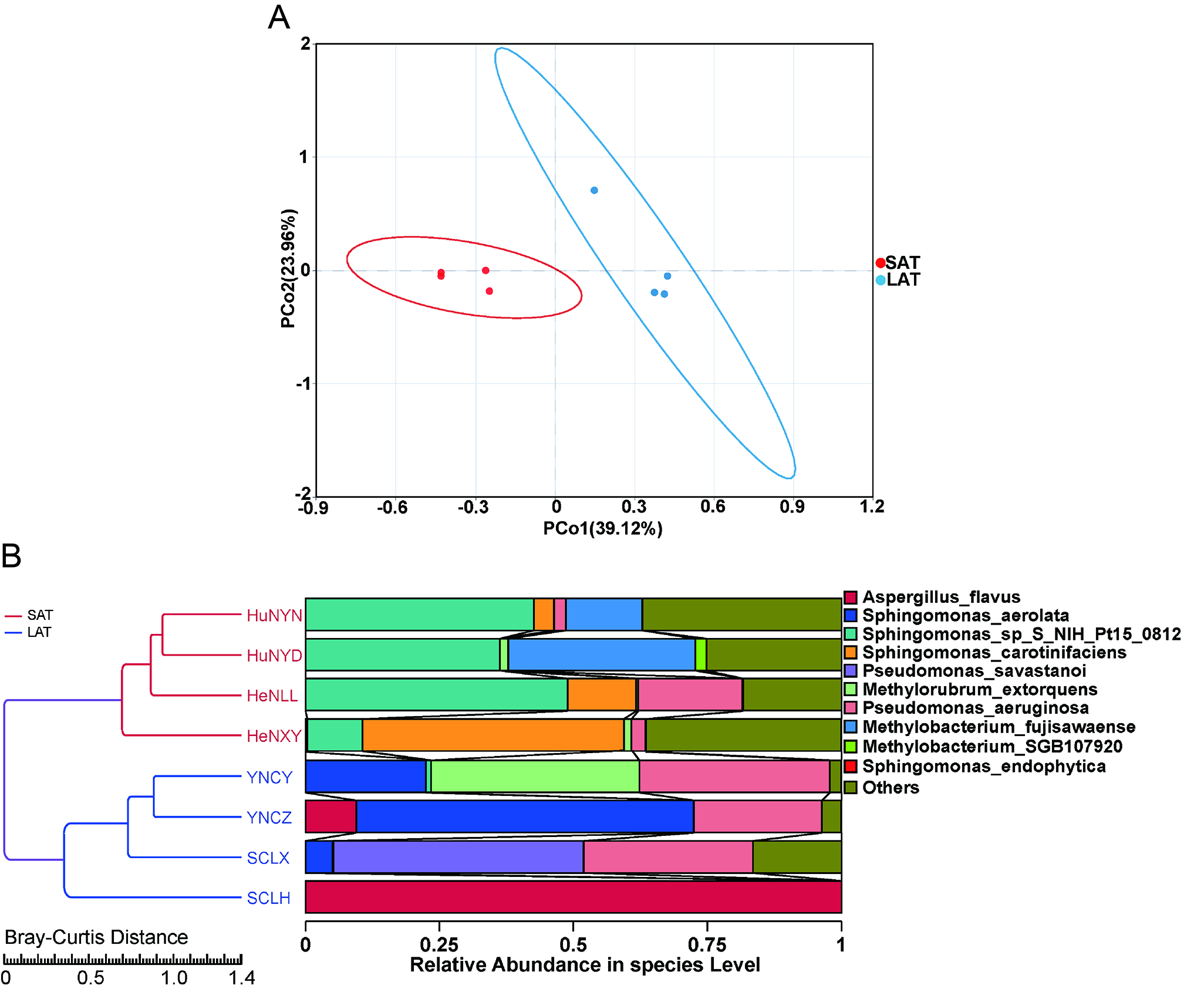

Supplement: Supplementary file 1 [file Image1.tif]

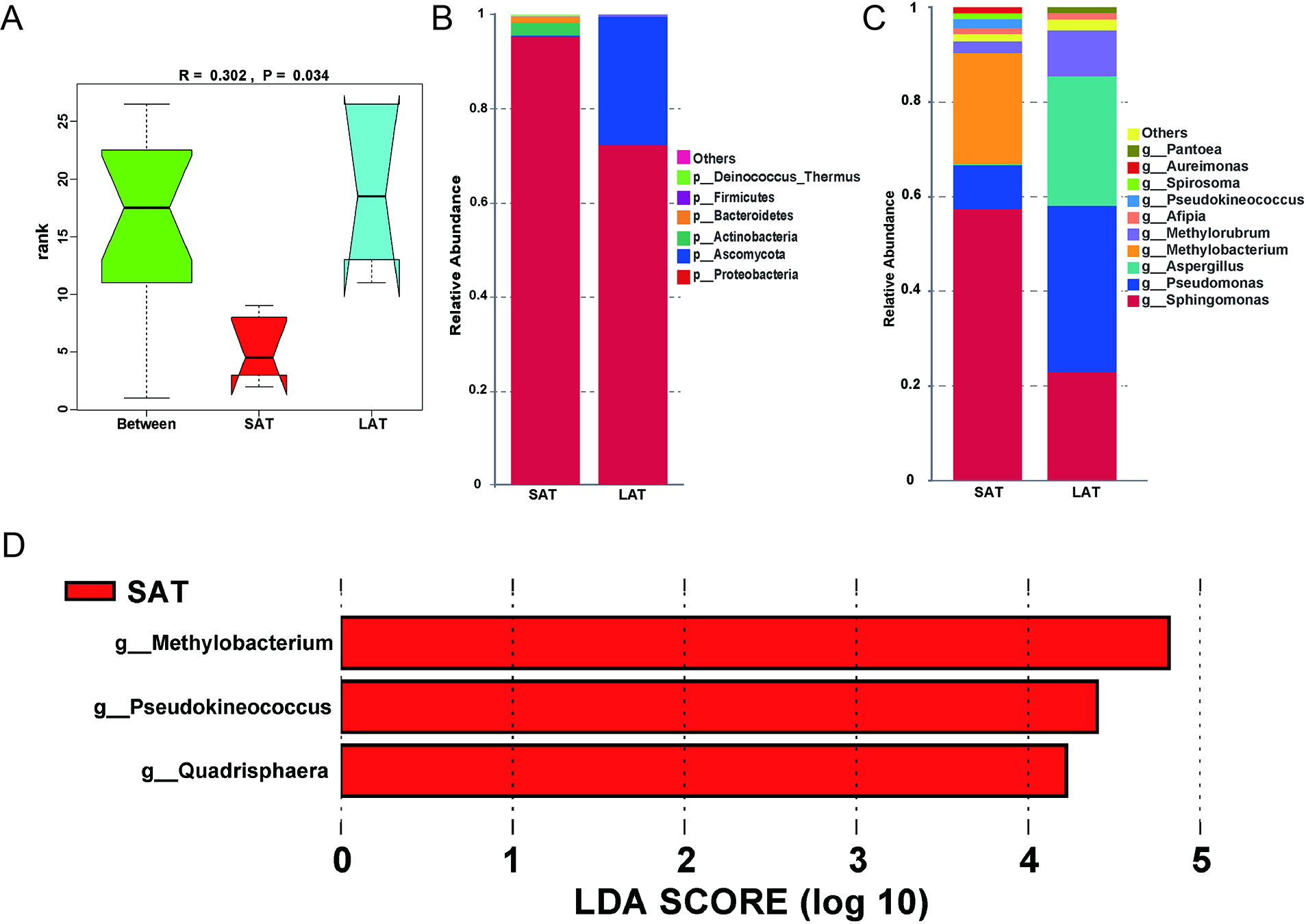

Supplement: Supplementary file 2 [file Image2.tif]
