# Supplementary material for: Integrated metabolomics and metagenomics reveal plant-microbe interactions driving aroma differentiation in flue-cured tobacco leaves
Source: Front Plant Sci. 2025 Jun 3;16:1588888. doi: 10.3389/fpls.2025.1588888 (PMC12170567; doi:10.3389/fpls.2025.1588888)
Supplement: Supplementary file 3 [file Table1.docx]

| Sample | ACE | Chao1 | Shannon (log2) | Simpson |
| --- | --- | --- | --- | --- |
| SAT | 54 | 54 | 3.465554685 | 0.839761 |
| LAT | 23 | 23 | 2.565885337 | 0.749087624 |

Table S1 Microbial α-diversity of aromatic tobacco leaves
